# Healthcare professionals in research (HPiR) Facebook community: a survey of U.K. doctoral and postdoctoral healthcare professionals outside of medicine

**DOI:** 10.1186/s12909-021-02672-1

**Published:** 2021-04-23

**Authors:** J. A. Deane, G. Clunie

**Affiliations:** 1 Sackler MSK LAB, Imperial College London, London, UK; 2 School of Health Sciences, University of Manchester and Manchester University NHS Foundation Trust, Jean McFarlane Building, Oxford Road, M13 9PL Manchester, UK; 3 Imperial College Healthcare NHS Trust, London, UK

**Keywords:** Allied Health Professionals, Nurses, Midwives, Clinical Academic careers, NMAHP, Peer support

## Abstract

**Background:**

Healthcare professionals outside of medicine (HCPs), including nurses, midwives and allied health professionals, are increasingly involved in research for patient benefit. Their challenge is to negotiate inter-professional or professionally isolated contexts. The aims of this study were to evaluate the ‘Healthcare Professionals in Research’ (HPiR) Facebook group (a self-directed and confidential peer support group for doctoral and postdoctoral HCPs) including engagement, the experiences of doctoral and postdoctoral HPiR members and to identify future career challenges using an on-line survey.

**Methods:**

The HPiR Facebook group was launched in May 2019. Five HCP Community managers (CMs) were trained in on-line platform curation, moderation and screening. An on-line survey was designed to capture data from HPiR members. A purposive sampling approach was applied. Respondents were required to be doctoral and postdoctoral HCPs and a registered member of the HPiR group. Respondents represented a range of healthcare professions, 79 % of whom had over ten years clinical experience. Membership growth and engagement was analysed. Descriptive statistics were used to present numerical data. Qualitative data were analysed thematically.

**Results:**

96 members were admitted to the group within the first month. All members were actively engaged with group content. 34/96 doctoral and postdoctoral HCPs completed the survey. Most members joined for networking (88 %) and peer support (82 %) purposes. Analysis of text responses showed difficulties in balancing a clinical academic career and highlighted the consequences of undefined clinical academic roles and pathways.

**Conclusions:**

Doctoral and postdoctoral HCPs value the opportunities that HPiR provides for peer support and connection with fellow HCPs. HPiR has the potential to strengthen research capacity, support research skill development and drive change within the clinical academic community. Clinical academic roles and pathways need to be standardised. The creation of opportunities beyond doctoral studies is a priority.

**Supplementary Information:**

The online version contains supplementary material available at 10.1186/s12909-021-02672-1.

## Background

Healthcare professionals outside of medicine (HCPs), including nurses, midwives and allied health professionals, are becoming increasingly involved in research for patient benefit. HCPs are ideally positioned to discover solutions for important clinical problems; adding a unique clinical perspective and driving change across a range of disciplines within the National Health Service (NHS).

For many years the NHS has recognised the value of supporting clinical research and clinical academic careers in order to improve NHS services, reduce healthcare costs and to provide the most effective care for patients. The National Institute for Health Research (NIHR) and Health Education England (HEE) have sought to develop the research capacity and capability of the NHS workforce through the design and development of bespoke doctoral and postdoctoral programmes for HCPs [1–3].

HCPs are inspired to undertake research and are keen to pursue clinical academic careers in order to improve patient care [4–6]. It is reported that doctoral and postdoctoral research provides personal satisfaction through improving patient outcomes, personal development and career progression, however, there are many barriers to overcome [4, 7, 8]. Common obstacles to clinical academic careers are reported to include funding, interference with work/life balance, a lack of clarity for the career path and support for clinical academics [9–12]. As HCPs cross the traditional divides between clinical and academic settings to engage in cutting edge research, the increasing challenge is to learn and thrive in new inter-professional or professionally isolated contexts.

Enablers facilitating clinical academic careers include mentorship, support, advice and guidance [9]. To support this the NIHR, the Council for Allied Health Professions Research (CAHPR) and respective professional societies provide free support and resources for clinical academics. To supplement this, the Healthcare Professionals in Research (HPiR) on-line group was developed to facilitate self-directed and confidential on-line peer support for doctoral and postdoctoral HCPs across the U.K. The group was developed by a multi-disciplinary steering group of doctoral and postdoctoral HCPs who were trained in the professional curation and moderation of the HPiR Facebook forum. The site was launched on May 18th 2019 and was subsequently officially supported by the CAHPR who promoted the site.

The objectives of this cross-sectional study were to review engagement with the HPiR group and to explore the experiences and perspectives of HPiR members with regard to doctoral research and the clinical academic pathway using an on-line survey.

## Methods

### Respondents

A purposive sampling approach was applied [13]. To be included in the study, respondents were required to be doctoral and postdoctoral HCPs and a registered member of the HPiR group. The survey was circulated electronically via online posts to HPiR group members in October-November 2019. Prior to completing the survey, respondents were able to read an information sheet and had the opportunity to ask questions by email. To improve response rate group members were also contacted individually by researchers using an online messenger service and several reminder posts were made on the online group.

### Design

 Ethical approval was granted by the Imperial College University Ethics Committee (ICREC reference 19IC5441). A cross-sectional, descriptive survey, comprising of four sections, was used to collate data regarding demographic details (Section 1: Clinical and Research Training and Education), reasons for joining and experiences of the HPiR group (Section 2: Healthcare Professionals in Research Group), experiences of doctoral research (Section 3: PhD) and future career perspectives, including the clinical academic role and pathway (Section 4: Post PhD and beyond) (See Additional file 1). Questions were a combination of multiple choice and free text responses. Questions were developed initially by JD using Qualtrics^XM^ software and refined in agreement with second author (GC). The questions were grouped into four main categories: Demographics, the HPiR online group, the doctoral process and the post-doctoral landscape. Survey questions were pre-tested for sense, items reduced and the survey piloted by five HPiR Community Managers (CMs). This confirmed face and content validity. Burns and Kho reporting guidelines for self-administered surveys of clinicians were used in the design and conduct of this study [14]. Both authors were involved in the design, verification and analysis process to maintain rigour.

#### Patient and public involvement

As CMs of the HPiR group, researchers (JD and GC), engaged with on-line HPiR members on a regular basis. In addition to the literature, impact stories from HPiR members informed the initial list of themes for discussion and evaluation. Feedback from two HCPs outside of the HPiR group were used to refine and validate the entire survey.

### Data analysis

Responses to the survey were recorded and summarised using Qualtrics^XM^ online software. Descriptive statistics were used to present numerical data (JD). The free text responses from the questionnaire were analysed within a computer assisted qualitative data analysis software (CAQDAS) programme (NVivo 12). Thematic analysis [15] was used to interpret the answers to free text questions and provide initial descriptive analysis. This was followed by an interpretive analysis of the responses to establish broad meanings from participants. Responses were analysed in this way by one researcher (GC) with a second (JD) verifying themes.

## Results

### Respondents

Ninety-six members were admitted to the group within the first month of launching HPiR. 100 % (96/96) of members actively engaged in the HPiR site through commenting (305 comments), posting (97 posts) or reacting (878 reactions) to group content.

Thirty-nine respondents provided informed consent to complete the online survey. Five responses were disregarded as they were incomplete at the time the survey closed (< 65 % complete). Thirty-four HPiR members completed the survey (35 % response rate (34/96)). The following findings are related to the remaining thirty-four participants.

### Clinical and research training and education

**Fig. 1:**
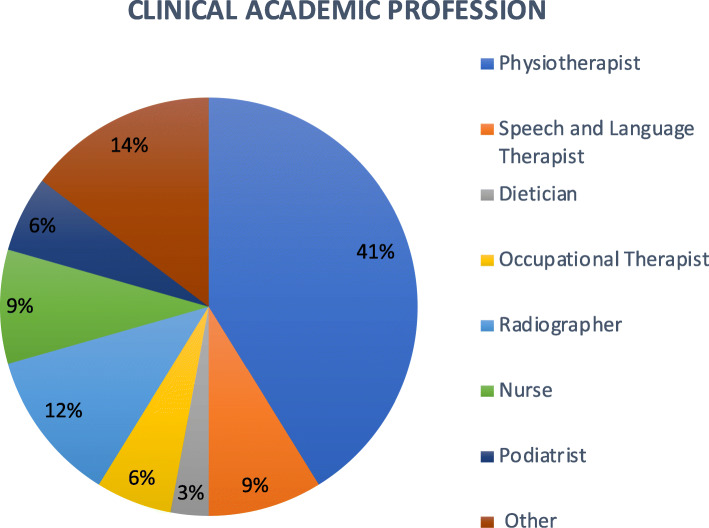
Clinical professions of clinical academic respondents

**Fig. 2:**
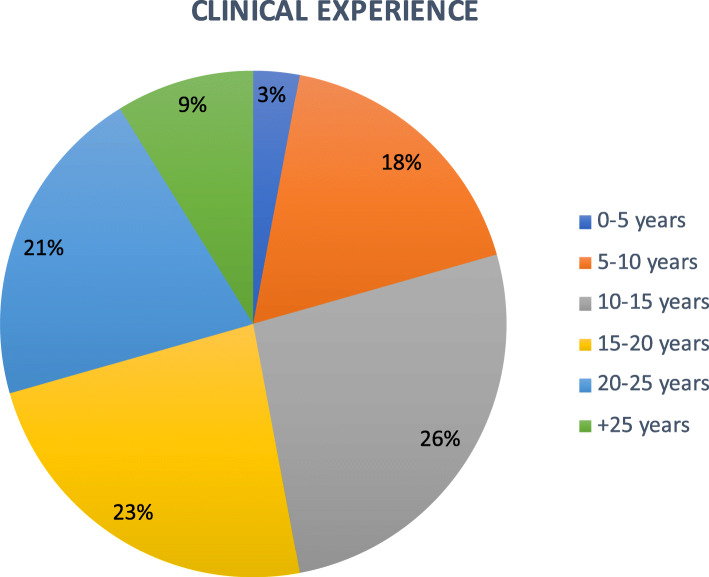
Clinical experience of respondents in years

Respondents reflected the multidisciplinary nature of the HPiR group, including Physiotherapy (41 %), Radiography (12 %), Speech and Language therapy (9 %), Nursing (9 %), Occupational therapy (6 %), Podiatry (6 %), Dietetics (3 %) and ‘other’ (Clinical Science, Orthoptics) (15 %) (Fig. 1), representing orthoptists and clinical scientists. Most respondents were undertaking doctoral studies (59 %) and the remainder were pursuing postdoctoral research (41 %). The majority of respondents worked within an NHS setting (65 %) and had over 10 years’ experience (79 %) working as a healthcare professional. 30 % of respondents had over 20 years post-qualification clinical experience (Fig. 2).

### Healthcare Professionals in Research Group

Respondents sought to join HPiR to facilitate networking (88 %, 30/34), peer support (82 %, 28/34) and for other reasons (12 %, 4/34), including ‘*giving back’* following difficult doctoral experiences, *‘to have a stronger voice’ and ‘to drive change in the clinical academic pathway’.* Respondents reported that the HPiR group had helped them in a myriad of ways; peer support (74 %, 25/34), reduced personal isolation (62 %, 21/34), encouraged writing practice (47 %, 16/34), alerted respondents to upcoming research events (41 %, 14/34), updated respondents regarding current research practice (35 %, 12/34) and assisted with problem solving (21 %, 7/34). Other ways (15 %, 5/34) in which HPiR had supported respondents included motivation through hearing about the successes of others and enhancing community through the establishment of connection with fellow healthcare professionals.

Most cited that peer support (74 %, 25/34) and reduced personal isolation (62 %, 21/34) were the primary ways in which the group helped. Others included encouraged writing practice (47 %, 16/34), being alerted to upcoming research events (41 %, 14/34), updated respondents regarding current research practice (35 %, 12/34), assisted with problem solving (21 %, 7/34) and ‘Other’ (15 %, 5/34) (hearing about other’s successes and enhancing connection).

Respondents believed that mentorship (71 %, 24/34), peer support (68 %, 23/34), related articles (38 %, 13/34), courses (24 %, 8/34) and books (18 %, 6/34) would have been of value in preparation for the doctoral process. The majority believed that access to HPiR would be beneficial for pre-doctoral students (56 %, 19/34). However, some respondents disagreed (12 %, 4/34) or were unsure (32 %, 15/34), which may reflect their preference for a specific doctoral and post-doctoral forum.

### PhD and post PhD and beyond

The majority of respondents worked within a supportive research and clinical environment (74 %, 25/34). However, 12 % (4/34) cited a lack of support, and 15 % remained ‘unsure’ (5/34). Although the survey was anonymous this lack of certainty indicated the complexity of an apparently simple question and may indicate a degree of reluctance to respond honestly.

48 % of respondents reported that their role, banding, grade or level would change upon completion of their doctorate. However, 52 % (17/33) believed that they would return to the same role they had left following their doctoral achievement, which was dissatisfying (82 %, 14/17).

42 % of healthcare professionals (14/33) felt that their departments are aware of the benefits of having a clinical academic as part of the team, however, some respondents indicated that their department was either not aware of the benefits (24 %, 8/33) or unsure of them (32 %, 11/33) (Table 1). In order to address this, respondents shared their knowledge and expertise with their teams; leading and supporting departmental research dissemination (26 %, 9/33), in-service training (26 %, 9/33), audits (15 %, 5/33) and journal clubs (6 %, 2/33). However, a significant majority of healthcare professionals (76 %) felt that there is a need for the clinical academic role to be defined more clearly.

In terms of future career goals, 85 % of respondents (29/33) would like to pursue a career in research and apply for postdoctoral funding (74 %, 25/33). However, 12 % of respondents (4/33) stated that it would be difficult to continue a career in research because of a lack of team support (Table 1).

**Table 1:** Respondent views of the PhD and Post PhD themes explored within survey

Theme	Items	Descriptors	Response (n)	Response rate (%)
***Section 3*** ***PhD***	Q12- On reflection, which type of support would you have valued in preparation for the PhD process? (you may select more than one answer)	Mentorship	24	71
		Peer support	23	68
		Courses	8	24
		Articles	13	38
		Books	6	18
		Other (e.g. networking, mentorship)	6	18
	Q13- Would it have been helpful to have been a member of a similar Facebook group pre-doctorally?	Yes	19	56
		No	4	12
		Unsure	11	32
	Q14- Are you currently working in a supportive research and clinical environment?	Yes	25	74
		No	4	12
		Unsure	5	15
	Q15 & Q16 invited qualitative responses only			
***Section 4*** ***Post PhD and beyond***	Q17- Will you return or have you returned to the same banding, level, grade or role after your PhD?	Yes	17	52
		No	16	48
		Unsure	0	0
	Q18- Are you satisfied with this? (please comment in the text box provided)	Yes	11	33
		No	14	42
		Other (no information offered)	8	24
	Q19- Is there a need for the clinical academic role to be defined more clearly?	Yes	26	79
		No	0	0
		Unsure	7	21
	Q20 & Q 21 invited qualitative responses only			
	Q22- Do you feel your department is aware of these benefits? (benefits of having a clinical academic as part of the team)	Yes	14	42
		No	8	24
		Unsure	11	33
	Q23- If yes, how have you made them aware? (you may select more than one answer)	In service training	9	26
		Journal club	2	6
		Audit	5	15
		Dissemination	9	26
		Other	12	35
	Q24- What are your future career goals? (you may select more than one answer)	I would like to continue in research	29	85
		I would like to apply for postdoctoral funding	25	74
		It will be difficult to continue with research as I do not have a supportive team	4	12

### Analysis of free text responses

Analysis of free text responses (Q10, Q11, Q15, Q16, Q20, Q21, Q25) confirmed that although the survey questions had been organised into specific headings (Section 1–4, see Design in Methods for further detail) the themes were overarching. The overarching themes included: (1) *Clinical-Academic Pathway; (2) Importance of Networks; (3) Struggle within the System.*

#### (1) Clinical‐academic pathway: “new approach required”

 Participants expressed the need for a clearer clinical-academic pathway for HCPs, more akin to the established model for medical colleagues.

*Introduce a system the medics have and allow staff members back into clinical career progression after PhD. (Physiotherapist)*

*I am not really a clinical academic. I personally do not think the NHS and NIHR has a pathway from Postdoctoral clinical academics. In the UK I couldn’t shake off the “just an AHP” mentality. (Radiographer)*

This was also identified as particularly important for HCPs as it offers an alternative career pathway within the NHS for example, instead of a managerial track.

There was an acknowledgement that the formal pathways available to HCPs e.g. NIHR Clinical Doctoral Research Fellowships had helped to develop the pathway, but that these remained limited and did not guarantee a clinical-academic future:

*It is fine now I am on a funding pathway but I’m about to fall off the edge of the fellowship cliff and I don’t know where I will be! (Physiotherapist)*

The lack of career and job security as a clinical-academic was a repeated concern by survey respondents. However, the HPiR group itself was seen to be a positive organisation reaffirming the potential to bring non-medical healthcare professionals together to help develop and embed the clinical academic pathway:

*Providing a space which is not currently available to us as AHPs, and to use this to be able to bring together NMAHPs [Nurses, Midwives and Allied Health Professionals] to have a stronger voice to really be able to drive change in the clinical academic pathway. (Physiotherapist)*

#### (2) Importance of network

The support network provided by the HPiR group was described as a positive resource, providing an opportunity for connection and sharing of experiences in a virtual environment.

*I think this ties in with the above categories but feeling of being connected to others completing similar activities and hearing their problems (and solutions) and their achievements. (Physiotherapist)*

Respondents described the isolation of their clinical-academic experience and the lack of a real-life support network; however, membership of the online group allows them to mitigate this.

*[I experience] Isolation as I work from home. The most valuable thing the site offers is for me a feeling of being connected to others doing something similar and somewhere I could ask questions. (Physiotherapist)*

Being able to ask questions of peers, and the potential for sharing knowledge and skills as part of the clinical-academic role were also regarded as a vital component of the online group, and of improving the clinical-academic experience.

*[I joined] to give back. Had such a tough journey during my PhD. Didn’t want anyone else to go through that. (Radiographer)*

There was also an acknowledgement that the HPiR group was a useful platform to share details of events, fellowships and skills-based learning relevant to members; with respondents making recommendations for further development and organisation of content.

*Keep up the good work. Maybe consider virtual meetings where members could have online video conversations (Nurse)*.

*I have found the links to useful guides etc. really helpful. So more of these would be great. (Radiographer)*

The network provided by HPiR was also seen to be a safe space to promote discussions about clinical-academic roles and the variability between different professions and organisations including the need for standardisation.

*The role itself will vary according to setting, service and local needs etc. However, I think NHS Trusts find it difficult to understand what a clinical academic is, and I think that justifies developing a broad role description/ framework that would help them to decide whether it is a role that they should invest in. (Physiotherapist)*

#### (3) Struggle within the system

The final theme that emerged from the free text responses related to the sense of struggle and frustration many group members experienced as clinical academics. Respondents described the challenges of trying to maintain a clinical role whilst also carrying out their research projects.

*Finding time to do everything, bringing my research and clinical roles together, feeling that I do both jobs inadequately, lack of understanding of the clinical academic role in the NHS. (Physiotherapist)*

*Balancing clinical workload with research activity and developing a new role within my department which has been met with barriers. (Radiographer)*

Free text responses reinforced a lack of understanding by managers of the benefits of having clinical academics in the NHS workforce with some post-doctoral group members reporting that they had to leave the NHS in order to further their careers.

*I was pushed out of clinical practice after my masters. “We can’t give you any research time, that’s not what we pay you for.“ Even now, nearly 9 years later, that still hurts. It affected my career for the rest of my life. I’ve never returned to the NHS. I’ve never been able to, even though I miss it. (Orthoptist)*

However, there was also an acknowledgment that clinical roles in the NHS are better paid than equivalent post-doctoral roles despite the possible sacrifice of research focus.

 Other responses were more positive about the struggle, observing that their role was to guide managers to see the value in clinical academic skills. The struggle for some was described as the need to advocate for the presence of clinical academics in teams to embed research in practice and promote excellence.

*It’s a tough gig but we are the filling in the sandwich, joining the hands of clinicians and researchers and making a Venn diagram of excellence. (Physiotherapist)*

## Discussion

The aims of this study were to use an on-line survey to review engagement with the HPiR group, explore the experiences of doctoral and postdoctoral HPiR members and to identify future career challenges. The growth of the HPiR group exceeded expectations within the first month with active engagement from all members. The survey evaluation reflects the views of experienced, multidisciplinary HCPs and demonstrates the potential value of confidential peer support within doctoral and postdoctoral clinical academic communities. This study identifies some key future challenges that are faced by this community relating specifically to the clinical-academic role and pathway.

### Engagement with the HPiR group

To our knowledge the HPiR group is one of the first on-line peer support forums for doctoral and postdoctoral HCPs in the U.K. Overall, HPiR was found to deliver what it set out to do, offer a confidential doctoral and postdoctoral peer support platform that facilitates peer support and reduce the isolation experienced by clinical-academics. However, the additional benefits for members have been transformative and motivating, enhancing skill development (such as problem solving and writing practice) and encouraging new connections between multi-disciplinary members across the U.K. that may not have been possible otherwise. Qualitative analysis reaffirmed that the connection between members was one of the most important features of the HPiR community. Overall, HPiR members consider this community a safe space in which to share knowledge, skills and to discuss important topics, such as clinical-academic roles and pathways.

It is acknowledged that the majority of respondents tended to be Allied Health Professionals, which may reflect the early official support and promotion of HPiR by the CAHPR in the U.K. In future, the same official support and promotion from nursing and midwifery councils could help to increase the representation of clinical academic nurses and midwives. Following this study, the HPiR group has successfully opened up admissions to a global community of pre-doctoral, doctoral and postdoctoral HCPs. Although further evaluation is required, the concept of HPiR appears transferable and valuable to international HCP communities.

### Experiences and perspectives of HPiR members with regard to doctoral research and the clinical academic pathway

It is encouraging to learn that most respondents work within supportive research and clinical environments. Consistent with previous research on the lived experience of clinical academics [10–16], it is a significant challenge to try to balance a clinical and academic role in an environments where the role is not understood or valued. This resulted in one respondent leaving the NHS. This finding is supported by evidence that the UK clinical-academic workforce seems to be declining with HCPs remaining purely clinical or moving completely into academia [12, 17]. Retaining high quality research staff is fundamental to maintaining critical mass, improving the quality of clinical services and ultimately enhancing patient outcomes [5, 12]. It is vital for HCPs, departments and services to understand the role and potential of clinical-academics more clearly [11]. In order to address this challenge and lack of clarity survey respondents are advocating for their roles using departmental education, facilitating dissemination, audits, in-service training and journal clubs.

Indeed, there appears to be an overwhelming need for both the clinical-academic role and pathway to be defined. This is supported by previous research where two of the most important barriers [11] to a clinical-academic role were lack of appreciation of the role and unclear infrastructure to the role. Respondents acknowledge that formal pathways, such as the NIHR Clinical Doctoral Research Fellowships [18], have helped greatly, however these pathways do not guarantee a clinical academic future. This lack of job security is a concern for HCPs and is reflected in the scarcity of opportunity for post-doctoral studies demonstrated in the literature [4, 9, 10]. Once fellowships end there are no guarantees regarding the next step, described by one respondent as falling ‘*off the edge of the fellowship cliff’*.

Over half of the respondents reported that they would return to the same banding, level or grade following years of doctoral or postdoctoral research. This is dissatisfying for HCPs and indicates a waste of funding resources and lack of appreciation for skills gained. It is suggested within the literature that organisational backing, flexible opportunities, funding and a coherent path will be required to change this [4, 19] so that doctoral work becomes the beginning of a research career and not a complete arrest of the research journey [20]. HCPs in this survey indicated that consideration of parity between HCP and medical career pathways would facilitate career progression and continuity. A broadening of opportunities through such schemes as the NIHR Integrated Academic Training (IAT) scheme [21] will help this, but a paradigm shift within for HCPs is necessary to embed this fully. HCPs have increasing opportunities to engage with a clinical academic career pathway with more funding opportunities available to them, particularly at the pre-doctoral and doctoral stages of their careers [4]. However for this to be a sustainable, long term career opportunity policymakers, funders and managers in academia and the NHS need to work alongside HCPs to allow for these careers to be supported at post-doctoral levels.

The majority of respondents would like to continue a career in research and demonstrate a willingness to apply for further funding, however the lack of clear clinical-academic roles, pathways, job security and in some cases, team support, it is difficult for some to continue to pursue an academic career. However, it is through multidisciplinary collaboration that a united and shared vision may be communicated and infrastructure improved [11].

In this respect, the HPiR group is viewed by respondents as a potential positive force for good, offering the potential to drive change so that HCPs *‘have a stronger voice’* in creating a pathway that is clear and fit for purpose.

### Limitations

The requirement for clinical academics to share their lived experiences was a strength of this study. The response rate (35 %) was over triple the average cited for similar on-line surveys (11 %) [22]. The power of this study may have been enhanced by incentivisation and undertaking the survey at a later stage in the development of the HPiR group. It is acknowledged that specific inclusion criteria and the need to understand the requirements of the group from the beginning limited the power of this study and may have biased the results, limiting the generalisability of results and further conclusions to be drawn in relation to specific professions. It is also acknowledged that this survey solely reflects the views of doctoral and postdoctoral HCPs. Furthermore, it is accepted that the NIHR pathway is not the only HCP clinical academic research route and that other routes are possible and could be examined in the future. Since the survey indicates that pre-doctoral HCPs may benefit from similar forums, it will be important to consider pre-doctoral support forums in addition to the recommended development and organisation of HPiR content and enhanced HPiR virtual connection opportunities in the future.

## Conclusions

Doctoral and postdoctoral HCP researchers actively engaged with the HPiR community and value the safe space it provides to discuss and connect with like-minded professionals. As a confidential peer support community of experienced HCPs, the HPiR group demonstrates the potential to strengthen research capacity, support research skill development and to drive change across the U.K. The HPiR group have identified the standardisation of the clinical-academic pathway and the creation of opportunities to continue in research beyond doctoral studies as a priority. Since undertaking this evaluation, the HPiR group now has over 300 members.

## Supplementary Information

**Additional file 1**

## Data Availability

Data that support the findings of this study are available upon reasonable request from the corresponding author.

## Abbreviations

HCPs: HealthCare Professionals outside of medicine
HPiR: Healthcare Professionals in Research
CMs: Community Managers
NHS: National Health Service
NIHR: National Institute for Health Research
HEE: Health Education England
CAHPR: Council for Allied Health Professions Research
ICREC: Imperial College University Ethics Committee
CAQDAS: Computer assisted qualitative data analysis software
