# Supplementary material for: Healthcare professionals in research (HPiR) Facebook community: a survey of U.K. doctoral and postdoctoral healthcare professionals outside of medicine
Source: BMC Med Educ. 2021 Apr 23;21:236. doi: 10.1186/s12909-021-02672-1 (PMC8067642; doi:10.1186/s12909-021-02672-1)
Supplement: Supplementary file 1 — Additional file 1 [file 12909_2021_2672_MOESM1_ESM.pdf]

Healthcare Professionals in Research

Consent Form for Participants Able to Give Consent
Full Title of Project: Healthcare Professionals in Research: an evaluation of an on-line doctoral and postdoctoral peer support community for U.K. Healthcare Professionals
Name of Principal Investigator: Dr Janet Deane
Co-investigator: Ms. Gemma Clunie
(Please select ALL boxes if you wish to proceed)

☐ 1. I confirm that I have read and understand the participant information sheet dated 4.7.19 version 1.0 for the above study and have had the opportunity to ask questions which have been answered fully. (4)

☐ 2. I understand that my participation is voluntary, and I am free to withdraw at any time, without giving any reason and without my legal rights being affected. (5)

☐ 3. I understand that my data will be automatically anonymised and I consent for the anonymised information collected to be used to support other research in the future, including those outside of the EEA. (6)

☐ 4. I give consent for my anonymised data to be used in future ethically approved studies. I give permission for my anonymised data to be sent to other organisations, including these outside of the EEA. (7)

☐ 5. I consent to take part in the above study. (8)

Section 1: Clinical and Research Training and Education

Q1 What is your current profession?

- Speech Therapist (1)
- Nurse (2)
- Radiographer (3)
- Physiotherapist (4)
- Occupational Therapist (5)
- Midwife (6)
- Podiatrist (7)
- Dietician (8)
- Clinical Psychologist (9)
- Other (please describe) (10)
____

Q2 Where are you based?

- Scotland (1)
- Wales (2)
- Northern Ireland (3)
- England (4)
- Other (please describe) (5) ____

Q3 Are you currently undertaking a PhD or Postdoctoral Training?

- ◯ PhD (1)
- ◯ Postdoctoral (2)

Q4 Are you currently working in the NHS?

- ◯ Yes (1)
- ◯ Other (please describe where you are working) (2) ____

Q5 If so, which Band are you?

- ◯ Band 5 (1)
- ◯ Band 6 (2)
- ◯ Band 7 (3)
- ◯ Band 8a (4)
- ◯ Band 8b (5)
- ◯ Other (please describe) (6) ____

Q6 How many years have you been qualified as a Healthcare Professional?

- ○ 0-5 years (1)
- ○ 5-10 years (2)
- ○ 10-15 years (3)
- ○ 15-20 years (4)
- ○ 20-25 years (5)
- ○ Other (please describe) (6) ____

Section 2: Healthcare Professionals in Research Group

Q7 How did you hear about the 'Healthcare Professionals in Research' (HPiR) Facebook group? (you may select more than one answer)

- ☐ Advertisement on Twitter (1)
- ☐ Advertisement on Facebook (2)
- ☐ Through CAHPR (3)
- ☐ Recommendation from colleague (4)
- ☐ Other (please describe) (5) ____

Q8 Why did you wish to join HPiR? (you may select more than one answer)

- ☐ Networking (1)
- ☐ Peer support (2)
- ☐ Other (please describe) (3) ____

Q9 How has the HPiR group helped you so far? (you may select more than one answer)

- ☐ Research practice updates (1)
- ☐ Problem solving (2)
- ☐ Peer support (3)
- ☐ Reduces isolation of the research experience (4)
- ☐ Keeps me abreast of research events (5)
- ☐ Encourages my writing practice (6)
- ☐ Other (please describe) (7) ____

Q10 Are there any aspects of the HPiR group that you find unhelpful?

- ○ No (please describe) (1) ____
- ○ Yes (please describe) (2) ____

Q11 Do you have any recommendations for further improvement? (provide brief comment below)

____

Section 3: PhD

Q12 On reflection, which type of support would you have valued in preparation for the PhD process? (you may select more than one answer)

- ▢ Mentorship (4)
- ▢ Peer support (5)
- ▢ Courses (please specify) (6) ____
- ▢ Articles (7)
- ▢ Books (8)
- ▢ Other (please comment) (9) ____

Q13 Would it have been helpful to have been a member of a similar Facebook group pre-doctorally?

- ○ Yes (1)
- ○ No (2)
- ○ Unsure (3)

Q14 Are you currently working in a supportive research and clinical environment?

- ◯ Yes (1)
- ◯ No (2)
- ◯ Unsure (3)

Q15 What are the challenges you face as a clinical academic?

____

Q16 Is there anything that can be done to improve this?

____

Section 4: Post PhD and beyond

Q17 Will you return or have you returned to the same banding, level, grade or role after your PhD?

- ◯ Yes (1)
- ◯ No (2)

Q18 Are you satisfied with this? (please comment in text box provided)

- ◯ Yes (1) ____
- ◯ No (2) ____
- ◯ Other (3) ____

Q19 Is there a need for the clinical academic role to be defined more clearly?

- ○ Yes (1)
- ○ No (2)
- ○ Unsure (3)

Q20 If so, how could should we go about this?

____

Q21 What do you feel the benefits are to having a clinical academic as part of the team?

____

Q22 Do you feel your department is aware of these benefits?

- ○ Yes (1)
- ○ No (2)
- ○ Unsure (3)

Q23 If yes, how have you made them aware? (you may select more than one answer)

- ☐ Inservice training (1)
- ☐ Journal club (2)
- ☐ Audit (3)
- ☐ Dissemination (4)
- ☐ Other (5) ____

Q24 What are your future career goals? (you may select more than one answer)

- ☐ I would like to continue in research (1)
- ☐ It will be difficult to continue with research as I do not have a supportive team (2)
- ☐ I would like to apply for postdoctoral funding (3)
- ☐ Other (4) ____

Q25 If there are any further comments that you have been unable to express as part of this survey please feel free to add your comments here.

- ○ I have no further comments (1)
- ○ I have further comments (please comment) (2)
____
